# Water filling and electric field-induced enhancement in the mechanical property of carbon nanotubes

**DOI:** 10.1038/srep17537

**Published:** 2015-12-01

**Authors:** H. F. Ye, Y. G. Zheng, Z. Q. Zhang, Z. Chen, H. W. Zhang

**Affiliations:** 1 State Key Laboratory of Structural Analysis for Industrial Equipment, Department of Engineering Mechanics, Faculty of Vehicle Engineering and Mechanics, Dalian University of Technology, Dalian 116024, P. R. China; 2 Micro/Nano Science and Technology Center, Jiangsu University, Zhenjiang 210013, P.R. China; 3 Department of Civil and Environmental Engineering, University of Missouri, Columbia, MO 65211, USA

## Abstract

The effects of water filling and electric field on the mechanical property of carbon nanotubes (CNTs) are investigated with molecular dynamics simulations. The simulation results indicate that the water filling and electric field could enhance the elastic modulus but reduce the Poisson’s ratio of the CNTs. As for the buckling behaviors, a significant enhancement could be observed in the yield stress and average post-buckling stress of the CNTs. In particular, the enhancement in the yield stress induced by the water filling and electric field could be even higher than that resulted from the solid filling. Moreover, a transition mechanism from the rod instability to shell buckling is shown to explain the nonmonotonic variation of yield stress, and the critical diameter can be tuned through filling the water molecules and applying the electric field. The present findings provide a valuable route for the optimized design and application of the nanoscale functional devices based on the water-filled CNTs.

Water-filled carbon nanotubes (CNTs) have been successfully separated and fabricated in laboratories in recent years based on autoclave treatment, density gradient ultracentrifugation, laser irradiation and so on^1^,^2^,^3^,^4^,^5^,^6^. As an incompressible fluid, water could provide a powerful support for the CNTs and consequently enhance the mechanical property of CNTs. In addition, it is well known that the water molecule is a typical polar molecule. Hence, the electric field may have an additional influence on the mechanical property of water-filled CNTs. Thus, it is anticipated that the mechanical property of CNTs could be modified through filling water molecules and applying electric field. In particular, a controllable mechanical property of CNTs may even be achieved via adjusting the filled water density and the electric field intensity.

Actually, the performance modification for the CNTs based on the filling has attracted much attention. However, many previous studies were mainly focused on the effect of the solid filling on the mechanical property of the CNTs. Wang *et al*. revealed that the critical buckling strain of CNTs would decrease firstly and then increase with the filling density of metal^7^, and the corresponding enhancement induced by the bimetallic alloys would be more obvious than that resulted from the pure metals^8^. Guo *et al*. found that the mechanical property and buckling process of CNTs could be influenced by the Au nanowire-filling^9^. By using molecular dynamics (MD) simulations, Jeong and Kim studied the effect of C_60_ fullerenes filling on the tensile, compressive and torsional behaviors of CNTs^10^. The results indicate that the critical compressive buckling load of the filled CNTs is increased by 19% as compared to that of the empty CNTs. In addition, there were a few investigations on the change in the mechanical property of the CNTs induced by the fluid filling. The simulations from Ni *et al*. revealed that the filling of CH_4_ and Ne could increase the buckling force of the CNTs by about 3% ~ 47%^11^. Through the experimental methods, Torres-Dias *et al*. found that the water filling induces an increasing difference between the radial breathing mode frequencies of water-filled and empty CNTs at elevated pressures^12^. Based on the water filling, our previous works explored the enhancements in the mechanical property of the (12, 12) CNT^13^ and further revealed the reversible stretching of the pre-strained (12, 12) water-filled CNTs controlled by the electric field^14^. In fact, for the different CNTs, the amount of the filling water molecules increases with increasing the diameter of CNTs. Hence, the mechanical property of water-filled CNTs may vary with the characteristic size of the CNTs. Moreover, as a nanoscale material with nearly perfect tubular structure and excellent mechanical property, CNTs are regarded as an ideal nanoscale carrier or container for nanoparticles, such as drug molecules, metal particles and so on^15^,^16^,^17^,^18^. Hence, the research on the mechanical property of the filled CNTs is rather important for the reliability and stability evaluation of these CNT-based systems. Furthermore, the CNTs are also considered as a promising candidate for the nanoscale functional devices due to their unique physical property. The filling could further improve the physical property of the CNTs and therefore expand their functionality to serve the need in various fields. As a novel material in laboratories, the water-filled CNTs possess great potential applications and have been of great interest. Therefore, the further investigation on the size-dependent mechanical property of water-filled CNTs could provide a better understanding on the reliability of drug delivery and nanoscale channel, and establish a solid foundation for the future development and applications.

In this paper, the mechanical property of water-filled CNTs is investigated via MD simulation of the compressive response, with a focus on the size effect. The five CNTs with different diameters are considered to examine the size dependence of the mechanical property. In addition, it has been proved that the electricity, the ubiquitous environment in biological molecules, could make an obvious effect on the water molecules inside the CNTs^19^,^20^,^21^. Therefore, the electric field is also taken into account in the MD simulation reported here.

## Results

**Figure 1:**
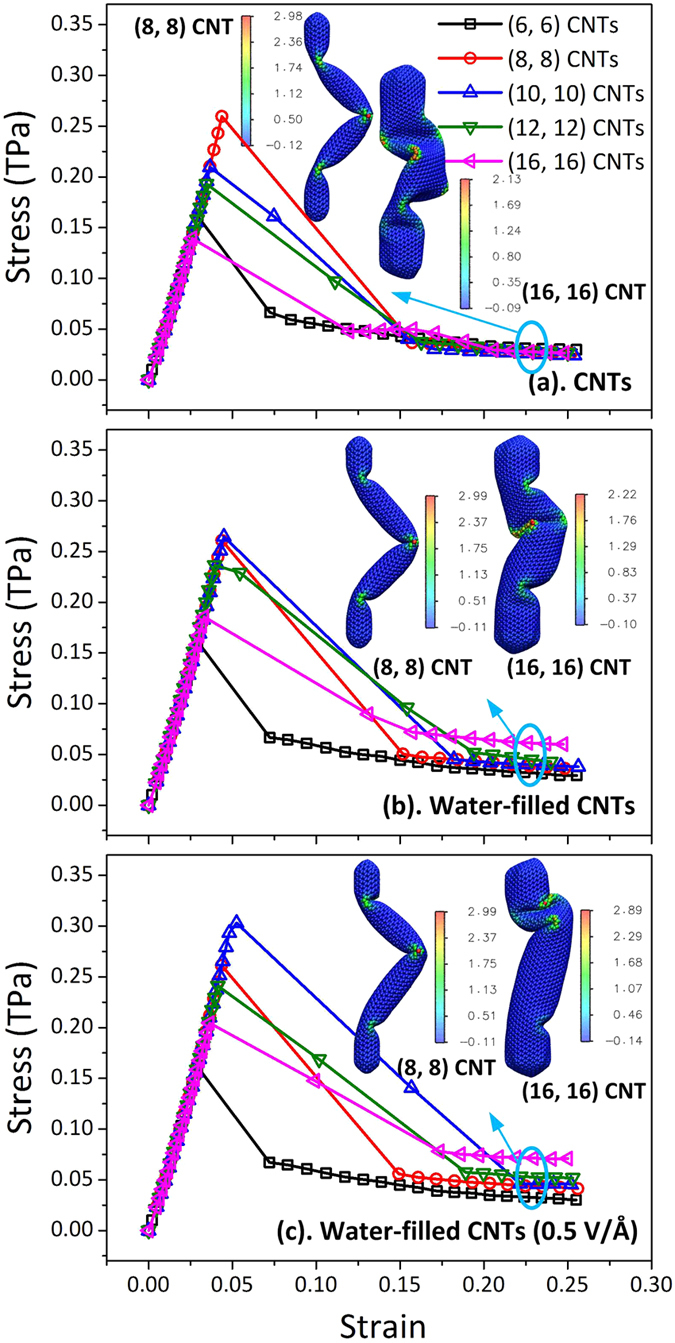
The stress-strain relationships. (**a**) the empty CNTs, (**b**) the water-filled CNTs and (**c**) the water-filled CNTs under the axial electric field with the intensity of 0.5 V/Å. The insets are the final buckling modes of the (8, 8) and (16, 16) CNTs. The color in the CNTs represents the distribution of the strain energy (eV) under the strain of about 23%.

Figure 1 shows the variations of the stresses with the strains of the empty CNTs, the water-filled CNTs and the water-filled CNTs under the electric field of 0.5 V/Å, respectively. In this work, the stress *σ* is calculated by the classical definition: *σ* = *F*/*A*, in which *F* is the spring force and *A* is the initial sectional area of CNTs. In the calculation of the sectional area, the thickness of CNTs is adopted as 0.66 Å^22^. From the figure, it can be seen that the stress linearly increases as the compressive strain increases in the initial elastic stage. Subsequently, when the strain reaches the critical buckling strain, the stress sharply decreases and the CNTs begin to buckle. For the buckled CNTs, the stress slowly decreases with the increase in the strain, and the variation range of the stress in this stage is rather small even though the strain keeps growing up to ~26%. The final buckling modes and the distributions of strain energy of the (8, 8) and (16, 16) CNTs are inserted in Fig. 1. The computational results reveal that the CNTs always present the asymmetric buckling mode in the final stage, which is similar to the common rod buckling. As compared to the (8, 8) CNTs, based on the global buckling deformation, some local wrinkles appear on the wall of the empty (16, 16) CNTs. For the water-filled CNTs, the CNTs look plump due to the filling of water molecules, and the local wrinkles on the wall of (16, 16) CNTs reduces. When the axial electric field is applied, the local wrinkles on the wall of (16, 16) CNTs almost disappear. Moreover, we can find that the high strain energy is always located on the positions of the large bending deformations along the CNTs. The average strain energies per carbon atom of the empty (8, 8) and (16, 16) CNTs under the strain of ~23% are 0.10 and 0.08 eV, respectively. After filling with water molecules, the corresponding average strain energies increase to 0.12 and 0.11 eV. Considering the electric field with the intensity of 0.5 eV/Å, a slight increase can still be observed for the average strain energies of the two CNTs. It is implied that under the same compressive strain, the water filling and electric field may speed up the compressive failure of CNTs, which is significant for the drug release and provides a reference point for the CNTs serving as a nanoscale fluid container.

**Figure 2:**
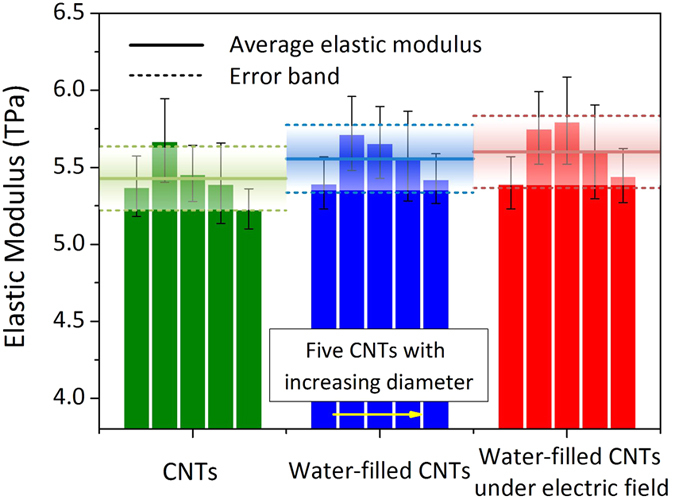
The elastic modulus of the CNTs in the three cases. The five columns in each case correspond to the elastic moduli of the (6, 6), (8, 8), (10, 10), (12, 12) and (16, 16) CNTs. The solid and dashed lines represent the averages and error bands of the five columns, respectively.

The elastic moduli of the empty CNTs, the water-filled CNTs and the water-filled CNTs under the electric fields of 0.5 V/Å are given in Fig. 2. The elastic modulus is defined as the slope of the linear stress-strain relationship in the whole elastic stage. The five columns in the three groups correspond to the elastic moduli of the (6, 6), (8, 8), (10, 10), (12, 12) and (16, 16) CNTs, respectively. Here, the elastic modulus and Poisson’s ratio are considered as the material properties which are independent on the characteristic size. For the empty CNTs, the average elastic modulus is 5.43  ± 0.21 TPa, which is consistent with 5.5 TPa as reported in the previous work based on the same CNT thickness^22^. As for the water-filled CNTs, the average elastic modulus is enhanced to be 5.56 ± 0.22 TPa, which is due to the additional support from the incompressible water. When the axial electric field is applied, the elastic modulus only has a small increase (5.60 ± 0.23 TPa). The results indicate that the filling of water molecules and the introduction of electric field could slightly enhance the elastic modulus of the CNTs.

**Figure 3:**
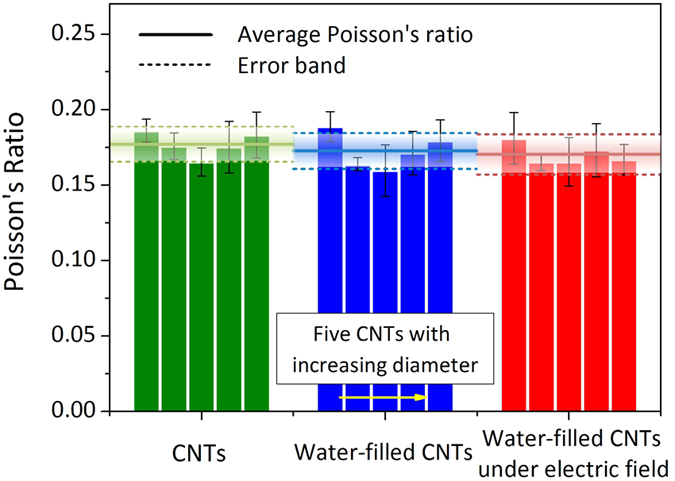
The Poisson’s ratio of the CNTs in the three cases. The five columns in each case correspond to the Poisson’s ratios of the (6, 6), (8, 8), (10, 10), (12, 12) and (16, 16) CNTs. The solid and dashed lines represent the averages and error bands of the five columns, respectively.

Fig. 3 displays the average Poisson’s ratios of the three types of CNTs, i.e., the empty CNTs, the water-filled CNTs and the water-filled CNTs under the electric field. Here, the Poisson’s ratio is calculated by the expression, *ν* = –*ε*_r_/*ε*_z_, where *ε*_r_ and *ε*_z_ are the circumferential and axial strains, respectively. The corresponding Poisson’s ratios for the three CNTs are 0.177 ± 0.012, 0.173 ± 0.012 and 0.170 ± 0.013, respectively. The present Poisson’s ratio of the empty CNTs is in the range of that obtained in the previous work (0.16 ~ 0.19)^22^,^23^,^24^. It can be seen that the water filling reduces the Poisson’s ratio of the CNTs, and the electric field could result in a further decrease in the Poisson’s ratio. The reason is because there has been a pre-strain in the circumferential direction for the water-filled CNTs as compared to the empty CNTs. Thus, for the water-filled CNTs, the further enlargement in the circumferential deformation becomes more difficult. Hence, it appears from the MD simulation that the water could offer a stronger suppression for the circumferential deformation than for the axial deformation.

**Figure 4:**
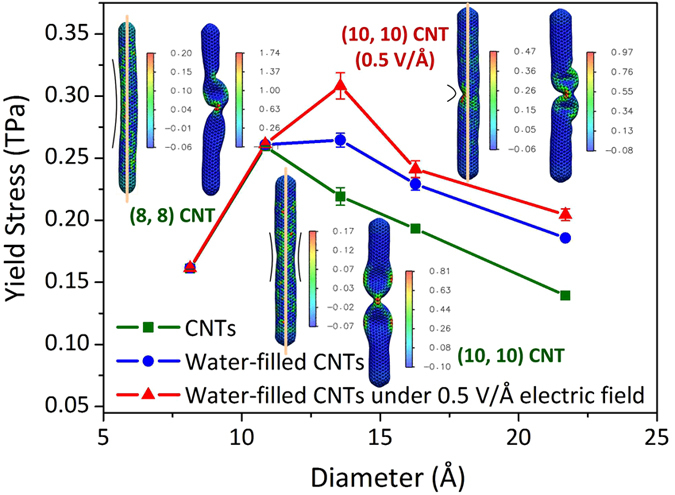
The yield stresses of the CNTs in the three cases under the compressive load. The insets illustrate the three representative configuration evolutions of the (8, 8) and (10, 10) CNTs in the initial buckling stage. The color on the wall of the CNTs represents the distribution of the strain energy (eV).

The yield stresses of the three types of the CNTs as a function of the diameter are plotted in Fig. 4. The yield stress reflects the critical value for the CNTs from the elastic to buckling deformations. For the empty CNTs (square samples), the yield stress has an increase for the (8, 8) CNT relative to the (6, 6) CNT, and then it decreases with the increase in the diameter. The nonmonotonic variation can be attributed to the different buckling mechanisms of the CNTs with different slenderness ratios. Though the final buckling modes of all the CNTs could be seen as the global instability, the initial buckling types, at which the stress just exceeds the yield stress, are completely different. The initial buckling mode of the small-diameter CNTs (the (6, 6) and (8, 8) CNTs) are the rod-like global buckling, as shown in the left two insets of Fig. 4. In the continuum theory, the yield stress in this case is in proportion to the square of diameter, i.e., 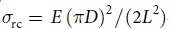, in which *E* is the elastic modulus, and *D* and *L* are the diameter and length of the CNTs^22^,^25^. As for the CNTs with larger diameter, the local buckling appears symmetrically and persists for several picoseconds, which is similar to the shell buckling, as shown in the lower two insets of Fig. 4. Based on the shell theory, the yield stress is in inverse proportion to the diameter and can be expressed as 
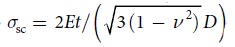
, in which *t* and *ν* are the thickness and Poisson’s ratio of the CNTs^22^,^25^. Based on the two equations as given above, a nonmonotonic trend of the yield stress can be obtained after identifying a transition diameter from the rod buckling to the shell buckling. As a result, the physics behind the present simulation results could be qualitatively explained although the average difference between the simulation results and the predictions from the continuum theory is about 20%. The turning point in the yield stress-diameter curve of the empty CNTs indicates that the transition diameter is in the range of 10.85 ~ 13.56 Å.

In addition, it could be observed that the water filling and electric field enhance the yield stress of the CNTs. For the (6, 6) and (8, 8) CNTs, the number of water molecules in the unit sectional area of the CNTs is very small. Hence, the enhancement in the yield stress induced by the water effect is not evident. As the diameter of the CNTs increases, however, the enhancement in the yield stress becomes obvious. As compared with the empty CNTs, the average enhancements of the last three CNTs induced by the water filling and the electric field of 0.5 V/Å are 23.1% and 36.6%, respectively. These enhancements are comparable to and even higher than those induced by the solid filling (5% ~ 33%), including C_60_, gold nanowires and metal^9^,^11^. Furthermore, the simulation results reveal that besides the enhancement in the yield stress, the water filling and electric field result in the change in the initial buckling mechanism of the (10, 10) CNT (the right two insets in Fig. 4), which implies the enlargement of the transition diameter of the rod and shell buckling deformations.

**Figure 5:**
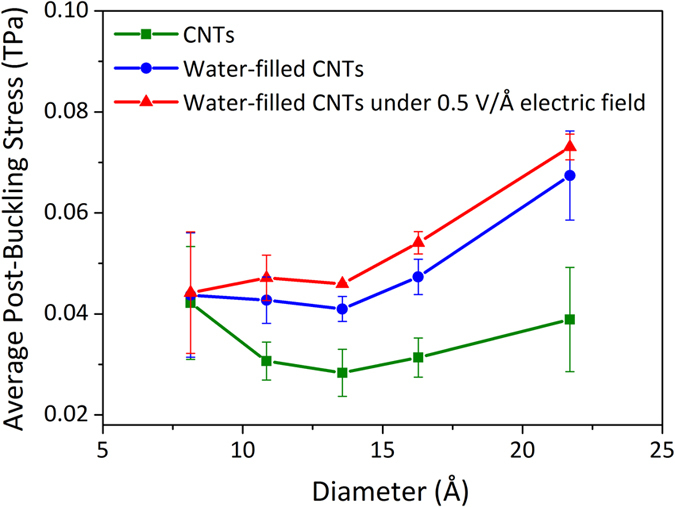
The average post-buckling stresses of the CNTs in the three cases under the compressive load.

It should be noted from the stress-strain curves that the buckled CNTs can still withstand a small compressive force. The resistant stress of the buckled CNTs, which is defined to be the post-buckling stress, only slightly decreases as the strain increases. The average post-buckling stresses of the buckled CNTs are shown in Fig. 5. The results indicate that the average post-buckling stress decreases firstly and then increases with the diameter of the empty CNTs. The average of the five average post-buckling stresses (five square samples) is about 0.034 ± 0.007 TPa. For the water-filled CNTs, the corresponding average value is increased by ~41.2% (0.048 ± 0.006 TPa). When the axial electric field with the intensity of 0.5 V/Å is applied, the average post-buckling stress of the five water-filled CNTs is increased to 0.053 ± 0.004 TPa, and the improvement in this case is about 55.9%. The present results demonstrate that the buckled CNTs could play a weak mechanical role, and water filling and electric field can enhance the mechanical performance of the buckled CNTs. It has been shown that the CNT can well maintain its structure even under a large deformation^26^,^27^. Hence, this weak and controllable mechanical property of the buckled CNTs may be significant for designing the restorable CNT-based devices to perform overloading tasks.

## Conclusion

In this work, the size-dependent mechanical property of water-filled CNTs is investigated by MD simulations. An electric field with the intensity of 0.5 V/Å is applied along the CNTs to further expand the influence of the internal polar water molecules. The simulation results reveal that all the CNTs in this study finally exhibit the global instability. Some local wrinkles can be observed on the wall of the CNTs with a large diameter, which can be reduced by the filling of water molecules and the introduction of electric field. Moreover, it is found that the water filling and electric field could slightly enhance the elastic modulus but reduce the Poisson’s ratio of the CNTs. For the buckling behavior, relative to the empty CNTs, the yield stress and average post-buckling stress have obvious enhancements due to the water filling and electric field. The high enhancement on the yield stress is even comparable to those induced by the solid filling, which may be ascribed to the incompressibility of water and the uniform distribution of water molecules inside the CNTs. Furthermore, as the slenderness ratio of the CNTs decreases, the initial buckling mode of the CNTs undergoes a transition from the rod instability to shell one, and the transition diameter can be changed through filling the water molecules and applying the electric field. In summary, for water-filled CNTs, the present research provides a valuable reference for designing the nanodevices based on this novel material. In addition, it is conceivable that the mechanical property of water-filled CNTs can be further changed via adjusting the water density and the electric field intensity, which may be utilized to fabricate the nanoscale functional devices.

## Methods

**Figure 6:**
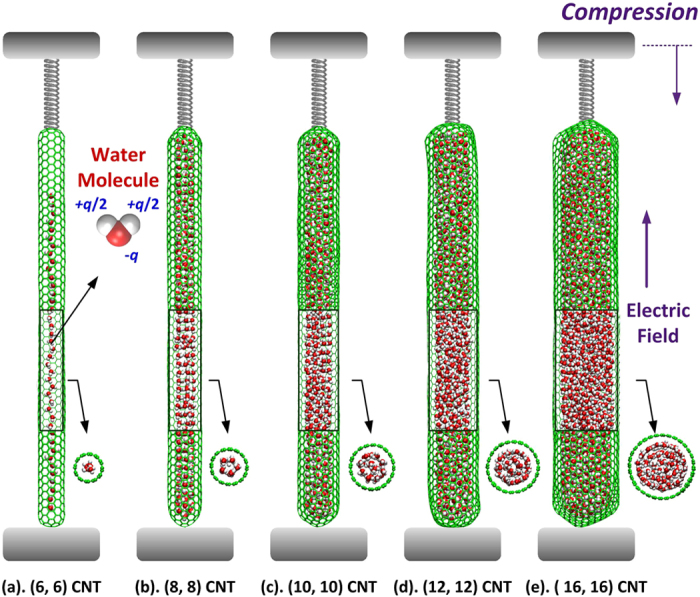
The computational models of the five water-filled CNTs. (**a**). (6, 6) CNT; (**b**). (8, 8) CNT; (**c**). (10, 10) CNT; (**d**). (12, 12) CNT and (**e**). (16, 16) CNT. The CNTs are colored in green, and O and H atoms of water molecules are in red and white, respectively.

The MD method is adopted to study the mechanical property of water-filled CNTs under the compressive load. Fig. 6 illustrates the computational models. Five capped armchair CNTs, i.e., (6, 6), (8, 8), (10, 10), (12, 12) and (16, 16) CNTs, are chosen as the water carriers. The bottom end of the water-filled CNTs is fixed on a rigid substance, and the top end is compressed by a spring. The spring is gradually compressed as 0.1 Å/ps and its length change is used to calculate the applied compressive force. Moreover, to examine the effect of loading rate, the stress-strain curves with a small spring velocity of 0.05 Å/ps is also extracted and presented in Supplementary Fig. S1 online. The consistent results demonstrate the rationality of the present loading rate. Without considering the boundary section, the effective lengths of the water-filled CNTs are about 100 Å. The filling density of water inside the CNTs is 1.0 g/cm^3^. The mechanical property of the CNTs is described by the reactive empirical bond-order (REBO) potential^28^. The water molecules are simulated by the TIP4P-EW model^29^, in which the bond lengths and angle degrees are constrained by the SHAKE algorithm to the initial values of 0.9572 Å and 104.52°, respectively. The atomic interactions between the carbon atoms of CNTs and the oxygen atoms of water molecules are calculated by the Lennard-Jones (LJ) potential, and the corresponding parameters are *σ*_CO_ = 3.28218 Å and *ε*_CO_ = 0.11831 = kcal/mol^30^. The particle-particle-particle-mesh method is adopted to compute the long-range Coulomb interactions between the polar water molecules. The cutoff distances of the LJ and Coulomb interactions are 12 Å and 10 Å, respectively. The position and velocity are updated through the canonical ensemble (NVT) with the integration time-step of 1 fs. The system temperature is maintained at room temperature (298 = K) by the Nosé-Hoover thermostat. In the present research, the effect of the electric field on the mechanical property of water-filled CNTs is also explored through applying an axial electric field along the CNTs. The electric field intensity is 0.5 V/Å, which is comparable to the average local electric field within the condensed phase of water^31^. Moreover, it can be found that this intensity is still in the range of high-intensity electric field in laboratories^32^,^33^,^34^,^35^. Initially, the relaxation times for the hollow and the water-filled CNTs are 100 and 300 ps, respectively. Subsequently, the spring is compressed by 1 Å and the system is equilibrated for 100 ps. To eliminate the influences of the initial configuration and velocity, the results below are the averages of six independent simulations for each case. The MD simulations are carried out by LAMMPS package^36^.

## Additional Information

**How to cite this article**: Ye, H. F. *et al*. Water filling and electric field-induced enhancement in the mechanical property of carbon nanotubes. *Sci. Rep.* **5**, 17537; doi: 10.1038/srep17537 (2015).

## Supplementary Material

Supplementary Information
