# Supplementary material for: Water filling and electric field-induced enhancement in the mechanical property of carbon nanotubes
Source: Sci Rep. 2015 Dec 1;5:17537. doi: 10.1038/srep17537 (PMC4664918; doi:10.1038/srep17537)
Supplement: Supplementary Information [file srep17537-s1.pdf]

Supplementary Information for:

Water filling and electric field-induced enhancement in the mechanical property of carbon nanotubes

H. F. Ye[1], Y. G. Zheng[1], Z. Q. Zhang[2], Z. Chen[1,3] & H. W. Zhang[1,*]

[1]State Key Laboratory of Structural Analysis for Industrial Equipment, Department of Engineering Mechanics, Faculty of Vehicle Engineering and Mechanics, Dalian University of Technology, Dalian 116024, P. R. China

[2]Micro/Nano Science and Technology Center, Jiangsu University, Zhenjiang 210013, P.R. China

[3]Department of Civil and Environmental Engineering, University of Missouri, Columbia, MO 65211, USA

Correspondence and requests for materials should be addressed to H.W.Z. (zhanghw@dlut.edu.cn)

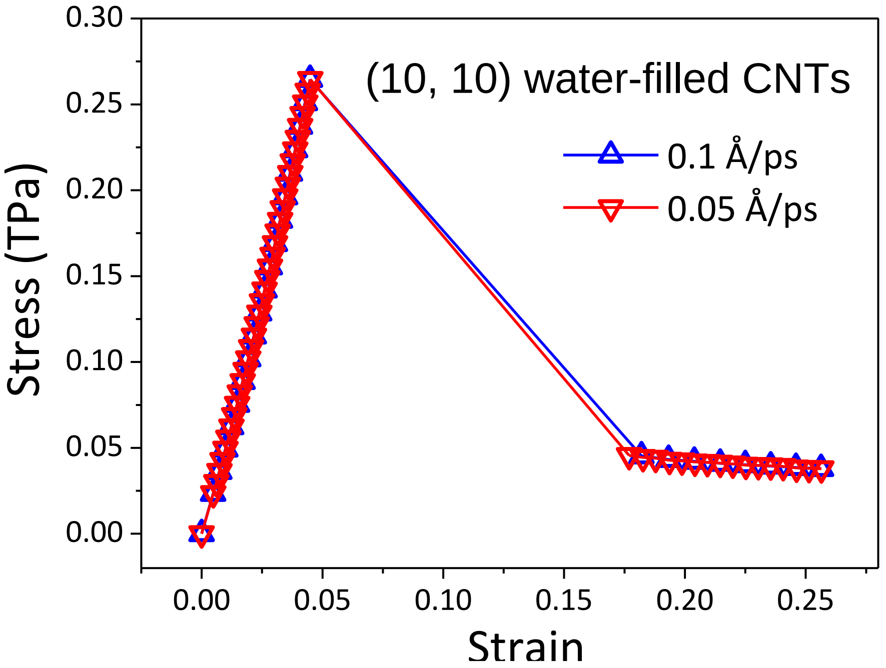

Supplementary Figure S1. The stress-strain curves of (10, 10) water-filled CNTs under the two different velocities of spring compression.
